# Physico-Chemical Characteristics of pH-Driven Active Film Loading with Curcumin Based on the Egg White Protein and Sodium Alginate Matrices

**DOI:** 10.3390/foods13091340

**Published:** 2024-04-26

**Authors:** Hanyu Li, Mengzhuo Liu, Xinyi Ju, Huajiang Zhang, Ning Xia, Jing Wang, Zhongjiang Wang, Ahmed M. Rayan

**Affiliations:** 1 College of Food Science, Northeast Agricultural University, Harbin 150030, China; lihanyu1004@126.com (H.L.); xianing1981@126.com (N.X.);; 2 Agricultural College, Suez Canal University, Ismailia 41522, Egypt; ammrayan@yahoo.com

**Keywords:** egg protein, curcumin, encapsulation, active film

## Abstract

The low solubility and stability of fat-soluble curcumin in water limit its application in active packaging. This study explored the use of a pH-driven method to investigate the preparation and enhancement of the performance of films loaded with curcumin in a matrix of sodium alginate (Alg) and egg white protein (EWP). In this study, the EWP, Alg, and curcumin primarily bind through hydrogen bonding, electrostatic interactions, and hydrophobic interactions. Compared to EWP films, the films loaded with curcumin through the pH-driven method exhibited enhanced extensibility and water resistance, with an elongation at break (EB) of 103.56 ± 3.13% and a water vapor permeability (WVP) of 1.67 ± 0.03 × 10^−10^ g·m/m^2^·Pa·s. The addition of Alg improved the encapsulation efficiency and thermal stability of curcumin, thereby enhancing the antioxidant activity of the film through the addition of 2,2-diphenyl-1-picrylhydrazyl (DPPH) and 2,2′-azinobis (3-ethylbenzothiazoline-6-sulfonic acid) (ABTS) radicals, which resulted in 106.95 ± 2.61 μg TE/g and 144.44 ± 8.89 μg TE/g, respectively. It is noteworthy that the detrimental effect of Alg on the color responsiveness of films containing curcumin has also been observed. This study provides a potential strategy and consideration for the loading of low water-soluble active substances and the preparation of active packaging.

## 1. Introduction

Increasing consumer awareness about food safety has led to a preference for foods with fewer additives. Consequently, researchers are relying more on packaging materials to keep food safe and prolong the shelf life of products [1]. However, traditional packaging materials, due to their lack of recyclability, have become a significant component of urban solid waste and are not aligned with contemporary eco-friendly principles [2]. Thus, the study of food packaging materials that are nontoxic, highly biocompatible, biodegradable, mechanically superior, safe, environmentally friendly, and economically viable has become a current research focus. For example, various bio-based and biodegradable polymer matrices have been used to develop food packages [3]. Natural macromolecular materials such as polysaccharides and proteins, which are formed into films through casting [4], pressing [5], electrospinning [6], etc., can create a network structure that impedes the migration of water vapor and reduces gas exchange between the interior and exterior of the film. Protein-based edible films are easily digested and absorbed by the human body [7,8]. Interactions such as hydrogen bonding, hydrophobic interactions, and disulfide linkages between protein molecules can form intermolecular cross-links, endowing films with excellent mechanical strength and barrier properties. Egg white protein (EWP), a widely available complete quality protein, has excellent film-forming and biocompatibility characteristics due to the presence of thiol groups and disulfide bonds [9]. It has already been widely used in active film matrices.

However, packaging films prepared solely from natural macromolecules fail to meet specific requirements, such as antioxidant properties, in food packaging. Hence, active packaging, a system that carries biologically active substances with antimicrobial, antioxidant, or freshness-indicating properties, has been proposed [10]. Compared to traditional preservation methods in which preservatives are directly added to food, active packaging enables the controlled release of natural active substances during storage, significantly slowing spoilage reactions originating from the food surface [11]. Curcumin, a generally recognized as safe (GRAS) food additive, has a broad spectrum of pharmacological activities, including antimicrobial, anti-inflammatory, antioxidant, and antitumor effects [12]. Both Gram-positive and Gram-negative bacteria are susceptible to its antibacterial activity [13], and its antioxidant activity is influenced mainly by β-diketone and phenolic hydroxyl functional groups [14]. Additionally, as a natural indicator, its color changes with pH variations above 8.0. Due to its favorable antioxidant and pH-responsive properties, curcumin has been extensively applied in active packaging film research. In previous studies, curcumin-incorporated active packaging films not only visually indicated changes in the internal environment of food packaging but also extended the shelf life of food products [15]. Currently, films with curcumin loaded in matrices such as gelatin protein, chitosan, and soy protein isolate have been proven to provide antioxidant, antimicrobial, and controlled release effects. By correlating film color changes with freshness indicators such as pH and total volatile basic nitrogen (TVB-N) levels, these films can be used to evaluate the freshness and preservation of perishable foods such as meat and seafood effectively [16]. However, the structure of curcumin has diketone and methoxy groups that are easily autoxidized, and the solubility and stability of curcumin in aqueous media are low, resulting in reduced bioactivity and film homogeneity [17]. Currently, strategies to increase solubility and stability of curcumin are mainly focused on protein loading and microencapsulation techniques using organic solvents such as ethanol and ethyl acetate to increase its solubility. However, the application of these organic solvents is limited due to safety and economic considerations for use in food packaging with direct food contact [18]. In addition, emulsion films with curcumin as filler are also used in the preparation of active films. Elevated temperatures and evaporation of water from emulsion films during drying may cause the oil phase of the emulsion to precipitate, forming a “pseudo-double film” with different properties on both sides [19], as the thermodynamic instability and the use of surfactants reduces the value of their application in food packaging. Unlike these methods, the pH-driven method, which does not require thermal processing, oil phases, and organic solvents, could improve the stability and dispersion of curcumin in protein solutions [20]. The pH-driven method has possible value for use in food packaging as the method utilizes only water as the sole solvent and the encapsulation of curcumin can be achieved by adjusting the pH using hydrochloric acid and sodium hydroxide. In this method, curcumin undergoes deprotonation and increases solubility during the alkalization stage, followed by maintaining its binding with the carrier through interactions like hydrogen bonding during the neutralization stage. Proteins undergo unfolding and refolding during this period, which helps curcumin to be encapsulated into the hydrophobic regions of the globular protein [21]. On the other hand, sodium alginate remains chemically stable throughout the pH adjustment process, with no significant changes in its structure [22].

Recent research on biomacromolecular active packaging films has indicated that single-matrix films have inherent limitations [23]. Films formed from a single protein exhibit good oxygen and oil resistance but suffer from poor mechanical performance, poor extensibility, and high-water permeability. Conversely, polysaccharide films demonstrate good gas barrier properties but lower water resistance and mechanical performance at higher humidities, limiting their application in the food industry [24,25,26]. Proteins and polysaccharides can improve the properties of blended films through interactions between two polymers (including covalent bonding via the Maillard’s reactions) and physical interactions (e.g., hydrophobic interactions, electrostatic interactions, hydrogen bonding, and van der Waals forces) [27,28]. For example, the interaction of alginate blended with wheat protein affects the microstructure, resulting in a denser microstructure. The blended films showed a greater barrier to UV light as well as greater elongation at break and water resistance than single alginate films [29]. With the help of the gel properties of EWP, the combination of EWP with polysaccharides induces the formation of a tighter three-dimensional network structure, which has the potential to improve the structural texture of the films as well as the defects of polysaccharide films, such as high hydrophilicity and ease of breakage, etc. Moreover, the dense network structure formed by protein-based and polysaccharide-based hydrogels also provides space for the transport of functional active ingredients, which greatly enhances the value of these films in food packaging applications [30,31]. However, the comprehensive modification in the antioxidant properties and color responsiveness of films resulting from the blending of different film-forming matrices has not been previously addressed. Particularly, in the context of the increasing development of composite matrix films, the impact of polysaccharide incorporation on the alteration of film color responsiveness merits investigation.

This study adopted a pH-driven methodology to prepare antioxidant-active packaging films with a blend of EWP and curcumin assisted by sodium alginate (Alg). The positive and negative effects of Alg blending, pH shifting, and curcumin on the physicochemical properties of protein-based films are dialectically analyzed. The antioxidant performance and color reactivity of curcumin after the addition of Alg were especially investigated in detail. The objective of this study is to improve the safety of active packaging in food preservation applications by dispensing with the oil phase and organic solvents, and to provide a promising pathway for the development of films loaded with hydrophobic actives.

## 2. Materials and Methods

### 2.1. Materials

EWP (3% moisture and 88% protein) was procured from Shan Song Biotechnology Co., Ltd. (Linyi, China). Curcumin (B20614, ≥98%), glycerol (B26015, ≥99%), and 1,1-Diphenyl-2-picrylhydrazyl (B25609, DPPH) were obtained from Yuan Ye Bio-Reagents Co., Ltd. (Shanghai, China). (±)-6-Hydroxy-2,5,7,8-tetramethylchromane-2-carboxylic acid (238813, Trolox) was obtained from Sigma–Aldrich (St. Louis, MO, USA). 2,2′-hydrazine-bis (3-ethylbenzothiazoline-6-sulfonic acid) (IA0010, ABTS) was purchased from Solarbio (Beijing, China). The water used in the experiments was distilled water (conductivity 1.53 μS/cm, pH 6.6). All other chemicals and solvents used were of analytical grade and distilled water was used to prepare all the solutions.

### 2.2. Film Formation

The preparation of EWP–curcumin–Alg nanocomplex dispersions via a pH-driven method was conducted following our previously described methodology with slight modifications [16]. Briefly, EWP dispersions were obtained by dispersing 9 g of EWP in 300 mL of distilled water and adding 9 g of glycerol with different concentrations of Alg (EWP: Alg = 8:1, 4:1, 2:1). After stirring at 45 °C for 3 h under magnetic stirring at 400 rpm, the dispersion was alkalized to pH of 11.0 with 2 mol/L NaOH followed by stirring of the mixture for 20 min. Subsequently, curcumin was added to the solution of EWP at a concentration of 2 mg/mL, after which the mixture was stirred for 20 min in an environment with no light source and under anaerobic conditions. The mixture was then neutralized to pH 7.0 with 2 mol/L HCl to obtain EWP–curcumin nanocomplexes. Subsequently, this mixture was centrifuged at 8000 rpm for 10 min to prepare the film-forming solution.

As a blank control for characterization, a film-forming solution without curcumin was prepared. All solution samples were evacuated in a vacuum dryer for 30 min to remove all foaming, and then cast onto a 200 mm × 200 mm × 6 mm PTFE plate, which was then dried at 50 °C for a continuous period of 12 h. The dried films were peeled off from the casting plate and conditioned at 25 °C and 50% RH at least for 48 h before further analysis. The nomenclature of all samples is summarized in Table 1.

### 2.3. Characterization of the Film-Forming Solution

The particle size and potential of the film-forming solutions were analyzed by a particle size and potential analyzer (Zetasizer Nano ZS90, Malvin Instruments Ltd., Malvern, UK). Samples were diluted to the appropriate concentration to avoid errors due to turbidity with distilled water adjusted to the corresponding pH.

Encapsulation efficiency (EE) and thermal degradation profiles of curcumin in solution were assessed by a consistent UV photometric method. The specific method was modified from that of Xiao et al. [32]. Specifically, the curcumin in solution was extracted and the protein was precipitated using a pre-configured mixture of ethanol and ethyl acetate extract, vortexed, and allowed to stand for 15 min. The supernatant was measured for UV absorbance at 425 nm and the curcumin content was calculated from the calibration curve. Notably, the thermal degradation curve was calculated by periodically measuring the absorbance of the solution supernatant during heating (50 °C, 16 h).

### 2.4. Characterization of the Physicochemical Properties of the Films

#### 2.4.1. Mechanical Performance

The thickness of the samples was determined with a liquid crystal thickness gauge (accuracy 0.001 mm, Delixi Instruments Co., Ltd., Leqing, China), and 10 points were averaged for each of the three parallel samples.

The elongation at break (EB) and tensile strength (TS) of the same size films (120 mm × 15 mm) were measured using a Byes-1001 universal testing machine (Bangyi Precision Gauge Co., Ltd., Suzhou, China). The test speed was set at 15 mm/min and the initial pitch was 50 mm.

#### 2.4.2. Optical Properties

The color indicators related to films (100 mm × 100 mm) brightness (L*), redness (a*), and yellowness (YI and b*) were measured by a CR-410 colorimeter (Konica Minolta, Tokyo, Japan) with a calibrated whiteboard (L = 97.17, a = −0.72, b = −0.17). In addition, the total color difference (ΔE*) was determined by the following equation:

(1) $$∆E*=\sqrt{{\left(L*-L\right)}^{2}+{\left(a*-a\right)}^{2}+{\left(b*-b\right)}^{2}}$$

The cut film samples (20 mm × 8 mm) were characterized for opacity values and UV blocking ability. Briefly, the samples were attached to the side of the cuvette and the samples were scanned for UV spectra using the transmittance mode. The specific parameters were: scanning range of 200–800 nm and scanning speed of 200 nm/min. The opacity and UV barrier of the samples were calculated by the following equations.

(2) $$Opacity\;value\left({mm}^{-1}\right)=\frac{A_{600}}{h}$$

(3) $$UV-A\;blocking\left(\%\right)=100-\frac{\int_{315}^{400}T\left(n\right)d\left(n\right)}{\int_{315}^{400}d\left(n\right)}\times 100$$

(4) $$UV-B\;blocking\left(\%\right)=100-\frac{\int_{280}^{315}T\left(n\right)d\left(n\right)}{\int_{280}^{315}d\left(n\right)}\times 100$$

where A_600_ is the absorbance of the film at 600 nm when the absorption wavelength is set to 600 nm and h is the average thickness of the sample, n is the absorption wavelength, and T (n) is the light transmittance at the current wavelength.

#### 2.4.3. Water Vapor Permeability (WVP) of the Films

The cup method was applied to measure the WVP, which was slightly modified from the method described by Xiao et al. [33]. A 200 mL cylindrical glass was sealed by a circular film with a diameter of 50 mm; the glass contained 5 g of CaCl_2_. The cups were then placed in a desiccator containing a saturated solution of KCl (relative humidity of 84%). The setup was maintained at 24 ± 1 °C for 7 days. After 7 days, the weight change in the CaCl_2_ solution was measured.

The WVP of the films was calculated using Equation (5):

(5) $$WVP\left(g\cdot m/m^{2}\cdot Pas\right)=\frac{h\times \Delta m}{\Delta t\times a\times \Delta W}$$

where Δt (h) is the holding time, h (mm) is the thickness of the sample, Δm (g) is the mass added to the cup, a (m^2^) is the area of the film that seals the cup, and ΔW (Pa) is the pressure difference between the inside and outside of the cup (1.998 kPa).

#### 2.4.4. Water Vapor Sorption (WVA)

The WVA of the films was determined according to the methods of Alizadeh-Sani et al. with slight modifications [34]. The film samples were completely dried in 0% relative humidity (saturated calcium nitrite solution) and then transferred to a desiccator maintained at 55% relative humidity and allowed to equilibrate for a period of three days. Subsequently, the WVA of the samples was calculated utilizing Equation (6):

(6) $$WVA\left(\%\right)=100\times {(m}_{e}-m_{0})/m_{0}$$

where m_0_ is the weight of films after equilibration at 0% relative humidity, and m_e_ is the weight of films after sorption of moisture.

#### 2.4.5. Water Contact Angle

A drop of 5 μL of distilled water was dripped on the surface of the films, and the surface hydrophobicity of the samples was assessed using the water contact angle as an indicator utilizing a Theta Lite video optical angle goniometer (Biolin Scientific Co., Ltd., Gothenburg, Sweden).

#### 2.4.6. Fourier Transform Infrared (FTIR) Spectroscopy

The FTIR absorption spectra of the films were recorded in the range of 400–4000 cm^−1^ based on an ATR-FTIR spectrometer (Nicolet IS50, Themo Fisher Scientific, Waltham, MA, USA). The number of scans was set to 64, and the resolution was 4 cm^−1^.

#### 2.4.7. X-ray Diffraction (XRD)

XRD curves of the samples were recorded using a powder XRD instrument with Cu-Kα radiation (D8 Advance, Bruker, Karlsruhe, Germany). The scanning speed was set to 5°/min and the scanning range was from 5° to 60° (2θ).

### 2.5. Functional Characterization of the Films

#### 2.5.1. Antioxidant Activity

The antioxidant capacity of the samples was judged by DPPH and ABTS free radical scavenging activities as previously reported [32].

The DPPH assay, briefly described, involved preparing an extraction solution by mixing methanol and distilled water in a 1:1 ratio. An amount of 0.1 g of the films was mixed with 20 mL of the extraction solution in the dark for 48 h. After centrifugation, the supernatant was collected. Two milliliters of the supernatant were reacted with 2 mL of DPPH methanol solution in an environment with no light source for 30 min. The absorbance of the post-reaction solution was measured at 517 nm, which was calculated using Equation (7):

(7) $$DPPH\;radical\;scavenging\;activity={(A}_{0}-A_{1})/A_{0}\times 100\%$$

For ABTS radical scavenging activity, ABTS reaction solutions were first produced as described by Lee et al. [35]. The film extracts were diluted 10 times with the reaction solution and reacted for 6 min in the dark. The absorbance of the sample at 734 nm absorption wavelength was recorded, and the ABTS radical scavenging activity of the films was calculated according to the following Equation (8):

(8) $$ABTS\;radical\;scavenging\;activity={(A}_{0}-A_{1})/A_{0}\times 100\%\;$$

where A_0_ is the absorbance of the blank solution and A_1_ is the absorbance of the prepared film.

#### 2.5.2. Volatile Amine Detection

The method for detecting volatile amines was modified from previous studies [36]. Ammonia, trimethylamine (TMA), and dimethylamine (DMA) were selected for the sensing tests. Briefly, 2 mL of different types of amines at different concentrations were added dropwise inside the Petri dish and the cut films (30 mm × 30 mm) were immediately attached to the inner side of the lid. The Petri dish was sealed with a sealing film and the reaction was carried out at room temperature for 1 h. The effect of molar concentration and type of volatile amine on the color indicators and visual observation of the film was determined. The color indicators were evaluated and calculated as in Section 2.4.

### 2.6. Curcumin Release

An amount of 2 g of films was dipped into 50 mL of 50% ethanol (a mixture of semisolid foods) and 95% ethanol (a mixture of fatty foods) in an environment with no light source. Periodically, 3 mL of the simulated solution was extracted for UV photometric assay (absorption at 425 nm), and the concentration of curcumin released was calculated using the standard curve in Section 2.3.

### 2.7. Statistical Analyses

Statistical analyses were conducted by IBM SPSS version 25.0 software via one-way analysis of variance (ANOVA) to analyze the significance of differences, followed by Duncan’s test for post hoc multiple comparisons. Differences were considered to be significant when *p* < 0.05.

## 3. Results and Discussion

### 3.1. Characterization of the Film-Forming Solution

**Figure 1:**
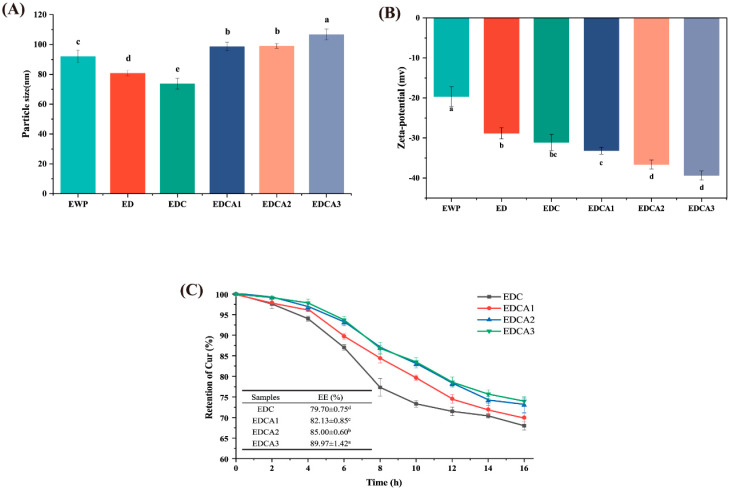
Effects of sodium alginate and curcumin on film-forming solution properties: (**A**) particle size, (**B**) ζ-potential, and (**C**) retention during 16 h of heat treatment and encapsulation efficiency (EE) of curcumin. Different letters (a–e) indicate significant differences (*p* < 0.05).

As shown in Figure 1A, compared to that of untreated EWP, the particle size of the pH-driven method significantly decreased, with ED particles measuring 80.83 ± 1.79 nm. This reduction is due to the exposure of negatively charged glutamic and aspartic acid residues under highly alkaline conditions, increased electrostatic repulsion between molecules, and disruption of aggregates stabilized by hydrophobic and electrostatic interactions [37]. The pH-driven method may also alter protein–protein interactions, causing the dissociation of certain subunits and leading to a reduction in protein molecule size [38]. The smaller particle size in the EDC (73.71 ± 3.61 nm) compared to that in the ED suggested that the hydrophobic nature of curcumin causes water expulsion from the protein, leading to tighter EWP structures and smaller particle sizes. Compared to ED and EDC, the addition of Alg resulted in a larger average particle size with a tendency to increase with increasing Alg concentration. A similar behavior was observed in the study by Ma [39], where the size of pea protein particles increased with an increasing Alg concentration. This was attributed to the involvement of electrostatic patch binding and hydrogen bonding interactions under near-neutral conditions [40].

The absolute value of the ζ-potential above 30 mV represents the relative stability of the protein or complex molecules, and within this range, electrostatic repulsion can maintain the dispersion of protein molecules and prevent aggregation [41]. As shown in Figure 1B, the ζ-potential of untreated EWP was −19.67 ± 2.45 mV. The pH-driven method leads to an increase in the absolute value of the ζ-potential of the EWP solution, which implies higher stability. This increase can primarily be attributed to the extensive exposure of hydrophobic groups during the alkalization stage of the pH-driven method, as well as incomplete folding after the neutralization stage. Consequently, some negatively charged amino acid side chains remain exposed on the protein surface [42]. The blending of Alg resulted in varying degrees of increase in the absolute value of the ζ-potential of the composite solution, with a further rise observed as the concentration of Alg increased. Notably, no significant difference was observed between the ζ-potential values of EDCA2 and EDCA3. This was attributed to the complete coverage of protein adsorption sites by Alg molecules, preventing further adsorption of excess Alg onto the protein surface [43].

As shown in Figure 1C, encapsulation of curcumin in colloidal particles was effective in protecting against particle degradation due to heat treatment. The addition of Alg increased the retention rate of curcumin in response to thermal treatment (*p* < 0.05). The highest concentration of residual curcumin was found in the EDCA3 film-forming solution, with a retention of 74.17 ± 1.08% after 16 h of heating, which was significantly greater than the 68.65 ± 0.57% retention rate in EDC. Moreover, curcumin was efficiently encapsulated using water as the only solvent through the pH-driven method, demonstrating its capability to be encapsulated effectively without the need for an oil phase. Notably, the EE of curcumin increased significantly after the addition of Alg (*p* < 0.05). With the increasing Alg concentration, EE gradually increased, likely due to the formation of a three-dimensional network structure between Alg and EWP, in which curcumin was captured within the network [44]. In summary, Alg can be used to assist the encapsulation of curcumin in EWP under pH-driven conditions and to reduce thermal degradation, making it promising for use in long heating and drying casting methods to prepare films.

### 3.2. Physical Performance of the Films

#### 3.2.1. Mechanical Performance

The thickness of the films is used as a basis for judging the other mechanical performance of the films. As shown in Table 2, ED films have significantly smaller thicknesses than EWP films (*p* < 0.05). This is because the pH-driven method obtains the protein structure of the molten globules, where the free side chains are exposed on the surface of the globular protein molecules, and this difficult stacking structure reduces the thickness of the film. Embedding curcumin reduced the thickness of the film further (*p* < 0.05). This result is similar to the report of Zhao et al. [45] who observed that the thickness of chicken protein-based films was significantly reduced after alkaline pH treatment compared to films prepared under neutral conditions. This observation can be attributed to the effective interaction between curcumin and the EWP matrix and the smaller particle size (in Section 3.1), which results in the formation of films with a more compact network. Notably, the addition of Alg did not significantly alter the thickness of the films. This observation is in agreement with the findings from Xiao et al. [32], who reported that a tight interpenetrating network structure was formed between the two matrices.

TS and EB are key indicators of the mechanical properties of films and represent strength and flexibility, respectively. The mechanical properties of the films are shown in Table 2. The addition of Alg significantly increased the TS of the films, mainly due to the formation of a dense network structure through covalent interactions between EWP and Alg, while curcumin was stably encapsulated within the structure, enhancing the tensile strength [46]. On the other hand, the pH-driven process significantly improved the EB of the films. This result may be due to the fact that more hydrophobic amino acids inside the protein were exposed and the weak interactions between these hydrophobic side chains changed the texture of the films [47]. It is noteworthy that the EDC films exhibited the highest EB, which can be attributed to both the enhancement of protein properties through the pH-driven method and the ability of curcumin to weaken intermolecular interactions among polymers [31,37]. With the addition of curcumin, curcumin enters the network structure formed between the proteins and binds to the proteins through hydrogen bonding and van der Waals forces, therefore weakening the interactions between the proteins. These factors collectively contribute to the increase in volume between the chains of protein and ductility of the films. Consistent with our expectations, given the known poor ductility of polysaccharide films, the blending of Alg did not enhance the EB of the films.

#### 3.2.2. Optical Properties

**Figure 2:**
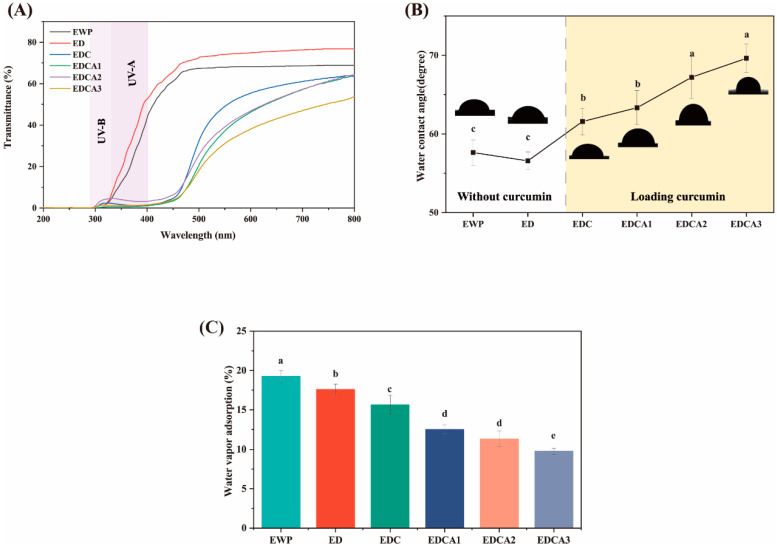
Water sensitivity and light barrier properties of the films: (**A**) light transmittance curves of films, (**B**) water contact angle, and (**C**) water vapor sorption (WVA). Different letters (a–e) indicate significant differences (*p* < 0.05).

Optical properties are crucial for determining the applicability of edible films in food packaging systems, as these properties affect the appearance of the packaged product and consumer acceptance. In Table 3, it is shown that the sole pH-driven process had almost no effect on the visible light transmission of the EWP films, suggesting that the pH-driven method primarily affects the microphase structure rather than the optical properties of films. The incorporation of curcumin and Alg significantly increased the opacity of the films, which was attributed to the covalent bonding between EWP and Alg, increasing the density of the film. A reduction in transparency is a disadvantage for food packaging that requires direct observation of the food state. This issue can be overcome in future research by improving the preparation method, reducing the thickness, or altering the proportions of additives. As shown in Figure 2A, films incorporating curcumin effectively absorbed UV-A radiation, which was mainly due to the diketone groups contained within curcumin loaded in the films [48]. This is consistent with Roy’s observation that the reduced light transmission of the composite film is mainly due to curcumin [49]. This was probably due to the ultraviolet absorption of the phenolic group of curcumin (1,7-bis-(4-hydroxy-3-methoxyphenyl)-1,6-heptadiene-3,5-dione). Compared with those of EDC, the films with added Alg absorbed more in the UV region, indicating stronger UV-blocking capabilities. This is similar to the phenomenon observed by Shahana Bishnoi et al. [29], who prepared films by combining sodium alginate with wheat proteins and found that the composite films absorbed more in the UV region and hence had lower transmittance as measured, indicating UV-blocking behavior. The blending of Alg with EWP resulted in the formation of a dense structure, which increased the EE of curcumin, thereby enhancing the blocking performance of the film in the UV-A region. All the films exhibited excellent UV-B blocking capabilities because of the spontaneous UV-B blocking ability of aromatic amino acids in EWP [50].

For the color indicators, the EWP and ED films have higher L* values and lower other color parameters, and the color difference with the calibrated whiteboard is very small. The above results indicate that the nearly transparent and colorless EWP films are excellent carriers for loading pH-sensitive dyes and exhibiting color changes. The loading of curcumin significantly reduced the L* values and increased the a*, b*, ΔE*, and YI values for the ED and EDC films (*p* < 0.05). Furthermore, the impacts of Alg concentration on the L*, a*, and b* values of the EWP films are shown in Table 3. After the blending of Alg, the L* values of the films significantly increased, while b* and ΔE* showed no significant changes. The YI followed a trend similar to that of a* value and was positively correlated with the total amount of protein-encapsulated curcumin in solution. Despite the close EE of curcumin in all three Alg-containing films, EDCA3 had the highest YI value, which was significantly different from that of the other Alg-containing films. This difference might be because the concentration of Alg was greater in the EDCA3 group than in the other two groups, and the abundant covalent binding in the films led to the formation of a more opaque and tightly packed network, which resulted in a more vibrant color of curcumin [51].

### 3.3. Water Resistance and Sensitivity

The WVP of the films is shown in Table 2. Typically, the protein particle size of the film-forming solution is positively correlated with the WVP value of the films [52]. Compared to EWP films, the sole pH-driven process reduced the granularity of EWP in the film-forming solution. Therefore, films prepared using the pH-driven method have strong water barrier properties (low WVP values). After the addition of curcumin, the WVP of several film groups was lower than that of pure EWP films [53]. This reduction might be due to the hydrophobic nature of the phenyl rings and the long carbon chains of curcumin, which increase the hydrophobicity of the films [54]. This improvement in barrier properties can also be explained by crosslinking between EWP and curcumin due to hydrogen bonding or hydrophobic interactions, which can reduce the free volume and increase tortuosity in the polymer network [55]. With the addition of Alg, the WVP gradually decreased as the concentration of Alg increased. This decrease might be due to the widespread interactions between Alg and proteins, resulting in tightly packed film molecules, a denser network structure, reduced film thickness, and improved barrier properties against water vapor. Compared to single-component films, composite films have smaller intermolecular gaps and a more complex network structure, making it more difficult for water molecules to permeate.

The contact angle is an important indicator of the hydrophilicity or hydrophobicity of the film surface. Typically, a smaller contact angle (θ < 90°) and larger WVA indicates a hydrophilic surface, while a larger angle (θ > 90°) and smaller WVA implies a hydrophobic surface [56]. As shown in Figure 2B,C, compared to those of films without curcumin, the EDC and EDCA films exhibited greater water contact angles and lower WVAs. The binding of curcumin to EWP reduced the formation of hydrogen bonds between the hydrophilic groups of proteins and water, thereby decreasing the water sensitivity of the films [57]. Dou et al. reported that the incorporation of polyphenolic active substances such as tea polyphenols into gelatin films reduced the hydrophilicity of the resulting protein films [58]. Compared to that of EDC, the water sensitivity of EDCA was lower. This improvement in the water sensitivity of the films might be due to the hydrogen bonding interactions between the hydrophilic groups (hydroxyl and carbonyl) of Alg in the film matrix and EWP, which reduces the number of water binding sites exposed on the surface of the films.

### 3.4. FTIR Spectra

**Figure 3:**
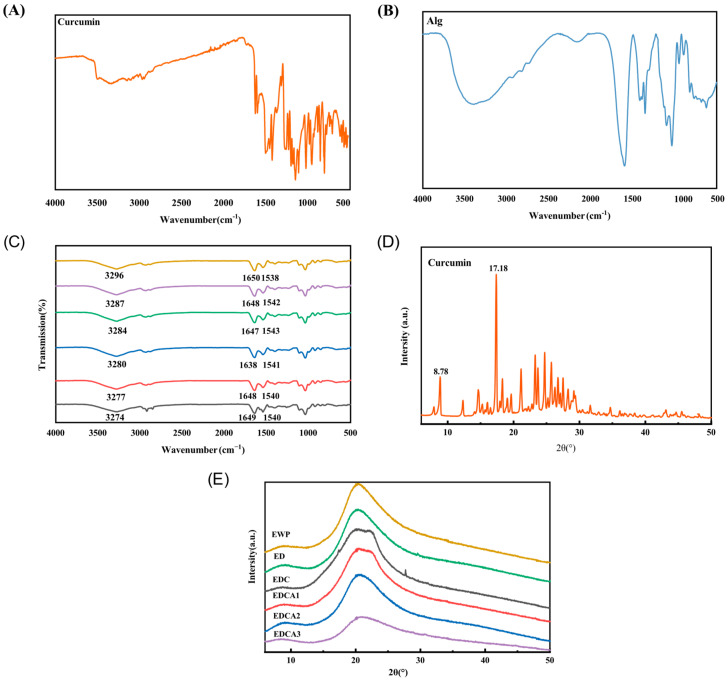
Effect of sodium alginate and curcumin on intermolecular interactions and molecular structure: (**A**,**B**) FTIR spectra of curcumin and sodium alginate, (**C**) FTIR spectra of thin films, (**D**) XRD patterns of curcumin, and (**E**) XRD patterns of films.

As indicated in Figure 3A–C, the spectra of EWP showed a characteristic peak at 3274 cm^−1^, which corresponds to the O-H stretching vibration, typically in the range of 3000–3500 cm^−1^ [59]. The position of the original FTIR characteristic peaks of the films in this range changed both after the pH-driven treatment of the proteins and the addition of curcumin, indicating that the pH-driven method caused the hydrogen bonds in the films to rearrange and thus affected the properties of the films. Furthermore, hydrogen bonding was the main driving force for the binding of EWP and curcumin in the film. After the addition of Alg, extensive hydrogen bonding cross-linking between EWP and Alg also occurred (3280–3296 cm^−1^), which was related to the presence of both Alg and EWP with a large number of hydrogen bonding donors (-OH) and acceptors (=O). Characteristic bands in the amide I and amide II regions of EWP near 1649 cm^−1^ and 1537 cm^−1^ were also observed. For EWP and ED films without added curcumin, almost identical characteristic peaks of amide I and amide II regions were observed, indicating that the pH-driven method has less effect on the secondary structure of the protein film. Upon the addition of curcumin, the position of the amide I band was blue-shifted due to hydrophobic interactions between the aromatic ring of curcumin and the amino acids of EWP. Upon the addition of Alg, the position of the amide I region was shifted in the opposite direction, implying that the interaction between Alg and EWP is predominantly hydrophilic. In addition to the shifting of the characteristic peaks, curcumin exhibited characteristic peaks mainly involving vibrations of the aromatic ring and the enol part, which mainly consisted of two regions of characteristic bands (1420–1630 cm^−1^ and 1110–1280 cm^−1^) [60]. In EDC films, the absorption peak disappears as curcumin is completely confined within the film matrix. In summary, a large number of hydrogen bonding interactions exist between EWP and Alg, and the binding between EWP and curcumin is mainly driven by hydrogen bonding and hydrophobic interactions. The above interactions play a key role in the formation of the film and are responsible for influencing the physicochemical properties of the film.

### 3.5. XRD Patterns

The crystallinity of a substance can be determined from its XRD pattern. Substances with sharp peaks are considered crystalline, while those without distinct peaks are considered amorphous. As shown in Figure 3D,E, the XRD pattern of curcumin showed multiple narrow and sharp crystalline peaks indicating its crystalline structure. On the contrary, all the films showed significant broad diffraction peaks centered at 2θ = 20°, indicating that the films are predominantly amorphous in nature. Notably, even in the curcumin-containing films, the sharp diffraction peaks of the curcumin crystals were almost undetectable, suggesting the successful encapsulation of curcumin in the film matrix using the pH-driven method [61]. Furthermore, with the blending of Alg, the diffraction peaks of curcumin crystals disappeared completely, and the intensity of the peaks decreased at 2θ = 20°. This can be attributed to the cross-linking between proteins and polysaccharides, which not only limits the thermal precipitation of curcumin, but also enhances the molecular mobility of the protein polymer chains and disrupts the alignment of protein molecules [62]. Additionally, a comparison of XRD patterns for films with varying concentrations of Alg revealed no significant differences. All films exhibited similar peak shapes, lacking any additional strong peaks. This suggests good compatibility between the highly concentrated and tightly bound Alg and EWP within the film matrix.

### 3.6. Antioxidant Properties

#### 3.6.1. DPPH and ABTS Radical Scavenging

**Figure 4:**
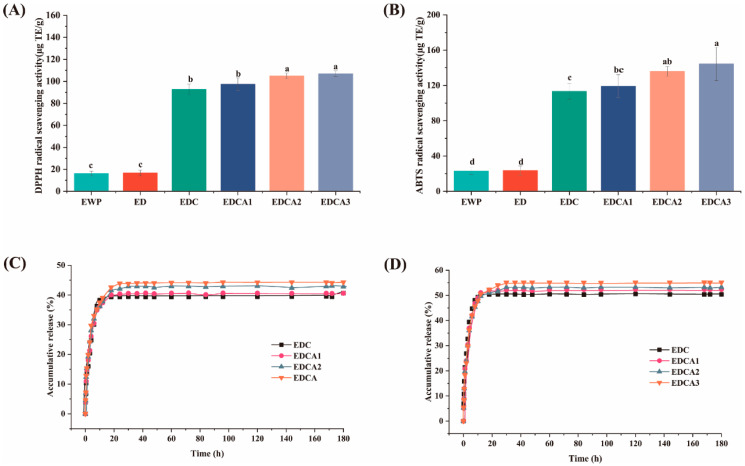
Antioxidant capacity of the films: free radical scavenging activities of (**A**) DPPH and (**B**) ABTS; curcumin release profiles in (**C**) semi-fat simulants and (**D**) fat simulants. Different letters (a–d) indicate significant differences (*p* < 0.05).

Curcumin has been proven to possess strong antioxidant properties. It can act as an antioxidant by interrupting chain oxidation reactions, donating hydrogen atoms, serving as a free radical acceptor, or chelating metals. As shown in Figure 4A,B, the DPPH and ABTS free radical scavenging effects indicate that curcumin-free EWP films have some low levels of antioxidant capacity. This antioxidant activity may be partly related to the antioxidant activity of some amino acids in the protein molecule, in addition to the small amount of lysozyme in EWP that may also provide some level of antioxidant capacity, which can act as electron donors to achieve antioxidant effects [63]. After the addition of curcumin, the antioxidant capacity of the films significantly increased, as indicated by an increase in the DPPH scavenging rate to 106.95 ± 2.61 μg TE/g and in the ABTS scavenging rate to 144.44 ± 8.89 μg TE/g. This difference is related to the phenolic hydroxyl groups and –CH2 groups in the structure of curcumin [64], and the increased water solubility of encapsulated curcumin can also greatly enhance its antioxidant activity. Compared to that of the films without Alg, the addition of Alg enhanced the antioxidant capacity of the films, which was related to Alg assisting EWP in improving the encapsulation rate of curcumin in the film. The experimental results indicate that the binding of Alg to proteins as a composite film matrix to load curcumin is a potential means to improve the antioxidant capacity of active packaging films. The combination of polysaccharides with proteins can also be used to encapsulate active substances with similar structures to maintain their activity [65].

#### 3.6.2. Curcumin Release

Given that the primary characteristic of bioactive films is the potential release rate of their active compounds, the release of curcumin from film samples into food simulants containing 50% or 95% ethanol was evaluated. As shown in Figure 4C,D, all the films exhibited similar release curves for both simulants, characterized by an initial burst release followed by a sustained slow release. The initial burst of release was attributed to the hydration of the polymer network upon contact with the simulant, which increased the mobility of the polymer chains and facilitated the release of curcumin.

As expected, all the composite films exhibited faster release in 95% ethanol, resulting in a shorter time required to achieve sustained release, and the percentage of curcumin released into 95% ethanol (fatty food simulant) was significantly greater than that in the semisolid food simulant. This difference could be attributed to the better solubility of curcumin in ethanol and its inherent lipophilicity, suggesting its potential for effective release in actual fatty systems rather than just simulants [66]. Notably, the blending of Alg effectively induced the sustained release of curcumin, as evidenced by the longer equilibrium times and greater cumulative release, ensuring the sustained color responsiveness and antioxidant activity of the film. This phenomenon related to the increased encapsulation rate, also ensuring that the films had higher antioxidant activity. In addition, we compared the fitting curves corresponding to various mathematical models for the curcumin release process. As shown in Table 4, the release pattern of curcumin in both simulants is close to the first-order model (R_2_: 0.9730–0.9860). The above results suggest that the release of curcumin is mainly limited by two parameters, including the real-time concentration of curcumin and the tightness of the film matrix. For the Ritger–Peppas model, the n-value represents the release pattern of the active substance in the film. The n-values of the films prepared in this study were all <0.2, which indicated that the diffusion mechanism of curcumin in the films was mainly Fickian diffusion, and the disintegration of the network structure of the films played little role in the process [67].

### 3.7. Color Responsiveness

**Figure 5:**
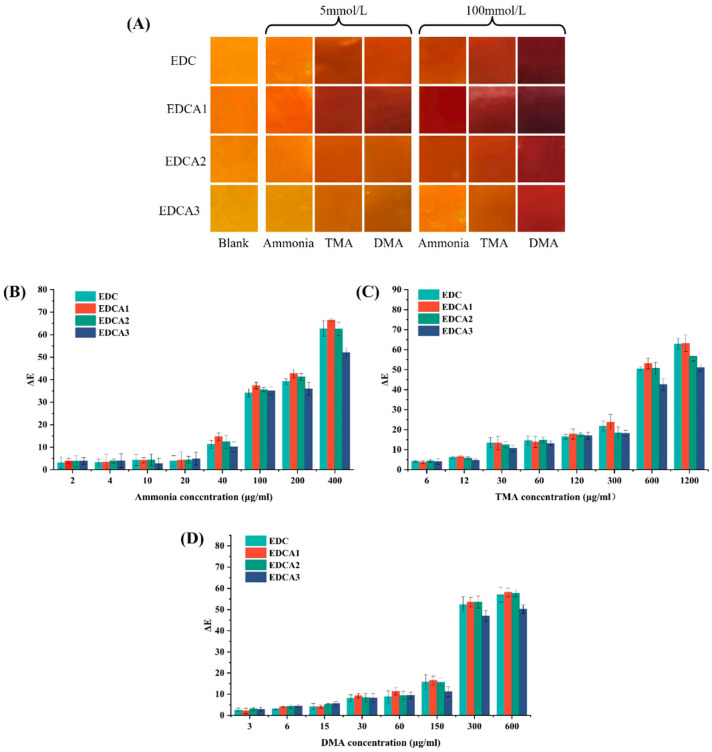
The ΔE* and corresponding colors using curcumin-containing films for the detection of volatile amines: (**A**) visual observation, (**B**) ammonia, (**C**) trimethylamine (TMA), and (**D**) dimethylamine (DMA).

Large amounts of TVB-N are produced during spoilage in foods, especially meat and aquatic foods. The response mechanism of the color of an indicator to volatile amines can be described as volatile amines initially binding with water on the hydrophilic film surface or surrounding environment, producing OH^−^. Subsequently, the phenoxide anion induces hydrogen transfer in the β-diketone chain of curcumin molecules [68], which exist in a keto-enol tautomeric form, leading to the red coloration of curcumin. Consistent with the expected results, all samples exhibited concentration-dependent color sensitivity to ammonia, TMA, and DMA (Figure 5A). The differential color sensitivity to different types of volatile amines is related to the pKa values of their conjugate acids, with the film’s apparent color showing the most pronounced response to DMA [16]. This is due to the fact that its conjugate acid has the highest pKa value of 10.75, presenting a variation from yellow to dark reddish brown. The films exposed to TMA and ammonia showed analogous color changes due to the close pKa values of their conjugate acids, which dissociate to an approximate degree in aqueous media. Therefore, the curcumin-loaded films exhibit diverse color sensitivities towards different types of volatile amines, enabling them to distinguish amines of certain concentrations. For the color parameters, the color sensitivity of all samples to ammonia, TMA and DMA was positively correlated with concentration. As shown in Figure 5B–D, with increasing concentrations of volatile amines, the ΔE* of all four film groups gradually increased. Contrary to the expected results, despite having higher EE for curcumin, EDCA films with added Alg demonstrated poorer volatile amine-color response, particularly with EDCA3 exhibiting the weakest color responsiveness. This is related to the increased tortuosity and density of the polymer network in the presence of Alg, as mentioned in Section 3.3. Another possible explanation is that Alg is a weakly acidic polysaccharide with a certain ion exchange capacity. When pH increases, the protons in −COOH can be released into the water, thereby buffering the pH change. Therefore, although the blending of Alg has the potential to enhance the antioxidant capacity and controlled release of the film, its impact on the color responsiveness appears to be detrimental.

## 4. Conclusions

This study prepared polysaccharide-protein films based on pH-driven method using EWP and Alg as raw materials. Firstly, the pH-driven method was used to increase the flexibility of protein molecules and the exposure of charged side chains, effectively encapsulating curcumin through hydrogen bonding and hydrophobic interactions. The blending of Alg improved the encapsulation rate of curcumin in the film-forming solution, thereby enhancing the activity of curcumin in aqueous media and further improving its antioxidant and controlled release properties. Additionally, blending Alg with EWP as the film matrix also enhanced the extensibility, visible light transmittance, and water barrier properties of the film, broadening its application range. However, the blending of Alg constrained the color responsiveness of the film, which requires further improvement in subsequent research. This study provides a new solvent-free and non-emulsification strategy for loading and stabilizing insoluble active substances into a film matrix.

## Figures and Tables

**Table 1:** Nomenclature rules based on film composition.

Sample Nomenclature	Film-Forming Liquid Formulations		
	EWP:Alg	Curcumin (%, *w*/*v*)	Method
EWP	0	0	Blending
ED	0	0	pH-driven
EDC	0	0.03	pH-driven
EDCA1	8:1	0.03	pH-driven
EDCA2	4:1	0.03	pH-driven
EDCA3	2:1	0.03	pH-driven

**Table 2:** Mechanical performance and water vapor permeability (WVP) of the films.

Films	Thickness (μm)	TS (MPa)	EB (%)	WVP (10^10^ g·m/m^2^⋅Pa·s)
EWP	126 ± 5 ^a^	2.72 ± 0.54 ^d^	62.68 ± 6.09 ^c^	1.41 ± 0.09 ^a^
ED	108 ± 5 ^b^	2.96 ± 0.43 ^cd^	96.44 ± 4.22 ^b^	1.35 ± 0.05 ^ab^
EDC	100 ± 4 ^c^	3.80 ± 0.18 ^bc^	108.86 ± 2.13 ^a^	1.33 ± 0.06 ^bc^
EDCA1	98 ± 2 ^c^	4.31 ± 0.32 ^b^	102.41 ± 2.10 ^ab^	1.31 ± 0.05 ^bc^
EDCA2	94 ± 4 ^c^	5.24 ± 0.63 ^a^	104.82 ± 0.97 ^a^	1.30 ± 0.04 ^bc^
EDCA3	94 ± 2 ^c^	5.68 ± 0.57 ^a^	103.56 ± 3.13 ^a^	1.25 ± 0.02 ^c^

Different letters (a–d) in the same column indicate significant differences (*p* < 0.05). TS: Tensile strength. EB: Elongation at break.

**Table 3:** Optical properties of the films.

Films	UV-A Blocking (%)	UV-B Blocking (%)	Opacity (mm^−1^)	L*	a*	b*	ΔE*	Yellowness Index (YI)
EWP	77.2 ± 50.48 ^b^	99.56 ± 0.64 ^a^	1.36 ± 0.11 ^d^	89.74 ± 0.71 ^a^	−2.97 ± 0.34 ^c^	9.70 ± 0.81 ^e^	11.74 ± 1.81 ^b^	18.43 ± 1.51 ^e^
ED	78.12 ± 0.60 ^b^	99.60 ± 0.90 ^a^	1.25 ± 0.12 ^d^	85.85 ± 2.86 ^b^	−0.61 ± 0.22 ^c^	5.66 ± 1.65 ^d^	14.31 ± 2.13 ^b^	18.27 ± 1.34 ^e^
EDC	99.03 ± 0.48 ^a^	99.58 ± 0.81 ^a^	2.34 ± 0.29 ^c^	76.04 ± 1.36 ^d^	32.37 ± 2.37 ^b^	73.93 ± 6.38 ^a^	69.19 ± 14.81 ^a^	106.81 ± 2.26 ^d^
EDCA1	99.21 ± 0.54 ^a^	99.61 ± 0.16 ^a^	3.17 ± 0.35 ^b^	81.68 ± 1.16 ^c^	33.03 ± 4.89 ^b^	63.92 ± 4.25 ^c^	62.44 ± 8.18 ^a^	118.41 ± 2.74 ^c^
EDCA2	99.38 ± 0.67 ^a^	99.70 ± 0.10 ^a^	3.35 ± 0.27 ^b^	82.04 ± 2.41 ^c^	36.70 ± 5.05 ^ab^	66.34 ± 3.825 ^bc^	70.12 ± 3.52 ^a^	123.97 ± 2.91 ^b^
EDCA3	99.35 ± 0.91 ^a^	99.66 ± 0.71 ^a^	3.68 ± 0.17 ^a^	82.30 ± 0.75 ^c^	41.16 ± 5.21 ^a^	70.40 ± 1.36 ^ab^	70.88 ± 4.34 ^a^	130.31 ± 4.08 ^a^

Different letters (a–e) in the same column indicate significant differences (*p* < 0.05).

**Table 4:** Correlation coefficients (R^2^) and release exponents (n, Ritger–Peppas) of curcumin release profiles in films fitted with different models.

Models	50% Ethanol (Semifat Food Simulant)				95% Ethanol (Fatty Food Simulant)			
	EDC	EDCA1	EDCA2	EDCA3	EDC	EDCA1	EDCA2	EDCA3
Ritger-peppas	0.7959	0.8205	0.8366	0.8395	0.7818	0.7974	0.8182	0.8242
n	0.16	0.16	0.16	0.16	0.13	0.15	0.16	0.17
First-order	0.9832	0.9796	0.9737	0.9730	0.9839	0.9860	0.9834	0.9860
Higuchi	0.2869	0.3017	0.3166	0.3224	0.2335	0.2715	0.2967	0.3131

## Data Availability

The original contributions presented in the study are included in the article, further inquiries can be directed to the corresponding author.
